# Beneficial Electrophysiological Effects of Rotigaptide Are Unable to Suppress Therapeutic Hypothermia-Provoked Ventricular Fibrillation in Failing Rabbit Hearts With Acute Ischemia–Reperfusion Injury

**DOI:** 10.3389/fphys.2021.726389

**Published:** 2021-09-13

**Authors:** Hui-Ling Lee, Po-Cheng Chang, Hung-Ta Wo, Hao-Tien Liu, Ming-Shien Wen, Chung-Chuan Chou

**Affiliations:** ^1^Department of Anesthesia, Chang Gung Memorial Hospital, Taipei City, Taiwan; ^2^Division of Cardiology, Department of Internal Medicine, Chang Gung Memorial Hospital, Taoyuan City, Taiwan; ^3^Chang Gung University College of Medicine, Taoyuan City, Taiwan

**Keywords:** ventricular fibrillation, optical mapping, ischemia-reperfusion, hypothermia, heart failure, connexin43

## Abstract

**Aims:** Whether therapeutic hypothermia (TH) is proarrhythmic in preexisting failing hearts with acute ischemia–reperfusion (IR) injury is unknown. Additionally, the effectiveness of rotigaptide on improving conduction slowing in hearts with IR injury is ambiguous. We investigated the electrophysiological effects of TH and rotigaptide in failing rabbit hearts with acute IR injury and determined the underlying molecular mechanisms.

**Methods and Results:** Heart failure was induced by right ventricular pacing (320 beats/min, 4 weeks). Rabbits with pacing-induced heart failure were randomly divided into TH (*n* = 14) and non-TH (*n* = 7) groups. The IR rabbit model was created by ligating the coronary artery for 60 min, followed by reperfusion for 15 min *in vivo*. Then, the hearts were excised quickly and Langendorff-perfused for simultaneous voltage and intracellular Ca^2+^ (Ca_i_) optical mapping. Electrophysiological studies were conducted, and vulnerability to ventricular fibrillation (VF) was evaluated using pacing protocols. TH (33°C) was instituted after baseline studies, and electrophysiological studies were repeated. Rotigaptide (300 nM) was infused for 20 min, and electrophysiological studies were repeated under TH. Cardiac tissues were sampled for Western blotting. TH increased the dispersion and beat-to-beat variability of action potential duration (APD), aggravated conduction slowing, and prolonged Ca_i_ decay to facilitate spatially discordant alternans (SDA) and VF induction. Rotigaptide reduced the dispersion and beat-to-beat variability of APD and improved slowed conduction to defer the onset of arrhythmogenic SDA by dynamic pacing and elevate the pacing threshold of VF during TH. However, the effect of rotigaptide on TH-enhanced VF inducibility was statistically insignificant. TH attenuated IR-induced dysregulation of protein expression, but its functional role remained uncertain.

**Conclusion:** Therapeutic hypothermia is proarrhythmic in failing hearts with acute IR injury. Rotigaptide improves TH-induced APD dispersion and beat-to-beat variability and conduction disturbance to defer the onset of arrhythmogenic SDA and elevate the VF threshold by dynamic pacing, but these beneficial electrophysiological effects are unable to suppress TH-enhanced VF inducibility significantly.

## Introduction

Cardiac ischemia–reperfusion (IR) injury-induced downregulation of voltage-gated sodium current (*I*_Na_) (Chang et al., 2018a) and alterations of gap junction uncoupling (De Groot and Coronel, 2004) are associated with conduction slowing and repolarization heterogeneities. Therapeutic hypothermia (TH) ameliorates oxidative injury induced during myocardial ischemia and reperfusion and interrupts the early stages of the apoptotic pathway during acute IR injury (Ning et al., 2002; Yamada et al., 2021). However, perturbations in electrophysiological properties during TH, including slow conduction velocity (CV), heterogeneous ventricular activations, and occurrence of spatially discordant alternans (SDA) (Hsieh et al., 2016), together might contribute to the proarrhythmic myocardial substrate and predispose hearts with acute IR injury to reentrant arrhythmias. The incidence of ventricular arrhythmia with hemodynamic instability was around 2.2% in patients with cardiac arrest due to ventricular fibrillation (VF) post-resuscitation undergoing TH (Hypothermia after Cardiac Arrest Study Group, 2002). Even if mild TH does not seem to significantly increase the occurrence of ventricular arrhythmias in patients with acute myocardial infarction undergoing percutaneous intervention (Noc and Aradi, 2017), a subpopulation of patients may be more prone to the development of ventricular arrhythmias (Yamada et al., 2021). Qureshi et al. (2019) reported that heart failure was associated with an increased mortality among cardiac arrest survivors treated with TH compared with patients without TH. Previous studies showed that heart failure enhances susceptibility to SDA, which markedly increases repolarization gradients and is associated with an increased incidence of inducible VF (Wilson et al., 2009). It is possible that TH enhances ventricular arrhythmias in failing hearts with acute IR injury.

Rotigaptide is a novel antiarrhythmic peptide analog that enhances gap junction conductance between ventricular myocytes, with little effect on membrane ionic currents (Xing et al., 2003). Hsieh et al. (2016) reported that it reduces repolarization heterogeneity, increases CV, and delays the onset of SDA to suppress TH-induced ventricular arrhythmias by enhancing cell-to-cell coupling in normal rabbit hearts. Hennan et al. (2006) reported that rotigaptide prevents IR-induced ventricular arrhythmias and reduces infarct size by increasing the presence of gap junctions in the area at risk. Theoretically, rotigaptide can prevent ventricular arrhythmias in hearts with acute IR injury undergoing TH. However, Tansey et al. (2006) demonstrated that IR-induced decrease in phosphorylated connexin43 (Cx43) does not affect CV in normal rabbit hearts after IR injury. Chowdhury et al. (2021) also observed that 7-day acute rotigaptide administration does not alter CV in a rat IR model. This may be attributed to the relatively large physiological reserve of gap junctions in the myocardium such that even a significant reduction in Cx43 is insufficient to result in functional conduction deficits in normal hearts. However, Akar et al. (2007) reported that gap junction reserve is significantly reduced by the increase in non-phosphorylated Cx43 (NP-Cx43) and lateralization of phosphorylated Cx43 in a pacing-induced heart failure rabbit model. We previously reported that CV in the IR zone (CV_IR_) is significantly slower than CV in the non-IR zone (CV_non–IR_), which is associated with the upregulation of NP-Cx43 in the IR zone in failing rabbit hearts (Chou et al., 2020b). On the basis of these findings, we hypothesized that rotigaptide plays an antiarrhythmic role in failing rabbit hearts with acute IR injury undergoing TH. In this study, we investigated the effects of TH and rotigaptide administration on cardiac electrophysiological parameters and vulnerability to VF by performing electrophysiological examinations and optical mapping studies. We also examined the underlying molecular mechanisms through immunoblotting to evaluate the remodeling of Cx43, Na^+^ channel, and Ca^2+^-handling protein expression in isolated failing rabbit hearts with acute IR injury undergoing TH.

## Materials and Methods

**FIGURE 1:**
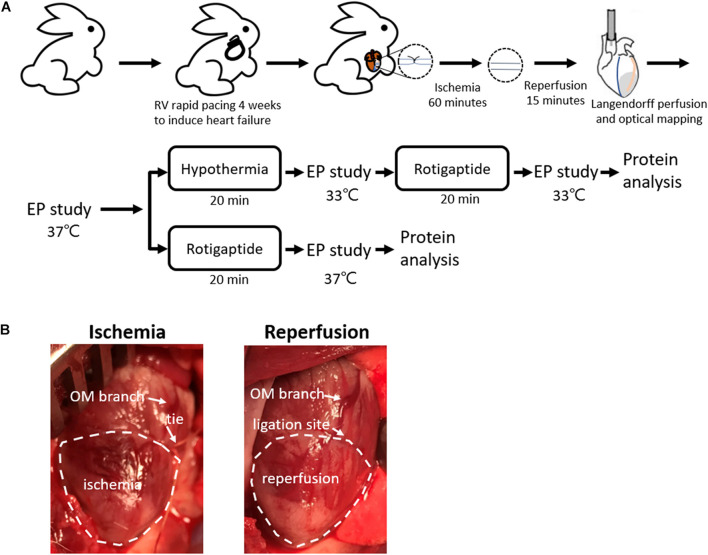
Rabbit ischemia–reperfusion (IR) heart failure model. **(A)** Protocol diagram. **(B)** Representative photos of IR model creation. Myocardial ischemia was induced by ligating the obtuse marginal (OM) branch of the left circumflex artery evidenced by darker color of the epicardium in the ischemic zone relative to the intact tissue, which was recovered by releasing the tie. EP, electrophysiological; LAD, left anterior descending artery; RV, right ventricle.

This research protocol was approved by the Institutional Animal Care and Use Committee of Chang Gung Memorial Hospital, Taiwan (Approval No. 2018090301) and conformed to the Guide for the Care and Use of Laboratory Animals published by the United States National Institutes of Health. Figure 1A shows the protocol diagram of the present study. New Zealand white rabbits (2.9–3.7 kg) were randomly divided into the following two groups: TH (*n* = 14) and non-TH groups (*n* = 7).

### Pacing-Induced Heart Failure

Rapid right ventricular pacing was used to induce heart failure as described previously (Chou et al., 2020b). Briefly, the rabbits were premedicated with intramuscular injections of zoletil (10 mg/kg) and xylazine (5 mg/kg). The surgical procedure was performed under general anesthesia with isoflurane (2%). An epicardial pacing lead was fixed on the right ventricle epicardium through a right lateral thoracotomy. The lead was then connected to a modified pacemaker (Adapta, Medtronic, Minneapolis, MN, United States). After 1-week recovery period, the heart was paced at a fixed rate of 320 bpm for 4 weeks to induce heart failure. Left ventricular (LV) function was assessed using echocardiography after 4 weeks of pacing.

### *In vivo* IR

An IR model was created as described previously (Chou et al., 2020b). Briefly, the rabbits were premedicated with intramuscular injections of zoletil (10 mg/kg) and xylazine (5 mg/kg), intubated, and anesthetized with isoflurane. When the rabbits were completely anesthetized and unresponsive to physical stimuli, the chests were opened through left thoracotomy. An obtuse marginal branch of the left circumflex artery was ligated midway between the atrioventricular groove and the cardiac apex for 60 min, followed by reperfusion. Occlusion of the obtuse marginal branch resulted in darker color of the epicardium in the ischemic zone relative to the intact tissue (Figure 1B). If spontaneous VF was induced, external defibrillation of 10–25 J was delivered.

### Langendorff Heart Preparation and Optical Mapping

After reperfusion for 15 min, the hearts were excised and Langendorff-perfused with 37°C Tyrode’s solution (composition in mmol/L: NaCl 125, KCl 4.5, MgCl2 0.25, NaHCO3 24, NaH2PO4 1.8, CaCl2 1.8, glucose 5.5, and albumin 50 mg/L in deionized water), and equilibrated with 95% O_2_ and 5% CO_2_ to maintain a pH of 7.4 for dual optical mapping studies. Rhod-2AM (Ca_i_ indicator, 5 μM, Molecular Probes, OR, United States; in 20% pluronic F-127 dissolved in dimethyl sulfoxide) and RH237 (Vm indicator, 1 μM, Molecular Probes; dissolved in dimethyl sulfoxide) were administered. The coronary perfusion pressure was regulated and maintained at 80–90 cmH_2_O. The hearts were illuminated using a solid-state, frequency-doubled laser light source (Millennia, Spectra-Physics Inc., Newport Corporation, Irvine, CA, United States) with a wavelength of 532 nm. Epifluorescence was acquired and filtered (715 mm for membrane voltage (Vm) and 580 nm for Ca_i_) with 2 MiCAM Ultima cameras (BrainVision, Tokyo, Japan) at 2 ms/frame temporal resolution and 100 × 100 pixels with spatial resolution of 0.25 × 0.25 mm^2^ per pixel. Motion artifacts were suppressed with blebbistatin (10 μmol/L; Tocris Bioscience, Minneapolis, MN, United States) (Chang et al., 2018b).

### Experimental Protocols

A bipolar catheter was inserted into the right ventricle for pacing at twice the threshold. The effective refractory period (ERP) was measured by giving a premature stimulus after 8 beats at a 400 ms pacing cycle length (PCL). Action potential duration (APD) and Ca_i_ alternans were induced by a dynamic pacing protocol reported previously (Chou et al., 2020b). VF inducibility was defined as the ability to provoke VF by a dynamic pacing protocol or extrastimulus pacing protocol (up to S_5_) (Chang et al., 2019). The VF threshold was defined as the longest PCL required to induce VF by dynamic pacing. Defibrillation using epicardial patch electrodes was performed for sustained VF (≥2 min).

Two thermostatic systems were connected in parallel to the Langendorff system. In the TH group, the induction of TH was performed by switching the thermostatic system and replacing the superfusate to 33°C after baseline studies. During cooling, the temperature of the left ventricle was monitored continuously with a thermometer implanted into the LV chamber *via* a left atriotomy. Electrophysiological studies were repeated after hypothermia for 20 min. Rotigaptide (300 nM) was then infused for 20 min (Hsieh et al., 2016), and electrophysiological studies were repeated under 33°C. In the non-TH group, rotigaptide was infused directly after the baseline studies.

### Western Blotting

Cardiac tissues were sampled from the non-IR and IR zones at the end of the mapping studies for protein quantification as previously described (*n* = 6 per group) (Chou et al., 2020b). Briefly, 50 μg of protein was loaded to each well in the SDS–PAGE gel for electrophoresis. The sample was then transferred onto a polyvinylidene difluoride membrane (Immobilon-P, EMD Millipore, Temecula, CA, United States). Primary antibodies against total Cx43 (1:500, GTX50571, Gene Tex, United States), NP-Cx43 (1:500, CX-1B1, Thermo Fisher Scientific, United States), Nav1.5 (SCN5A, 1:500, ASC-005, Alomone, United States), calmodulin-dependent protein kinase II (CaMKII, 1:2000, #3357, Cell Signaling, United States), pThr287-CaMKII (CaMKII-p, 1:1000, PA5-35501, Thermo Fisher Scientific, United States), phospholamban (PLB, 1:2000, #14562, Cell Signaling), pThr^17^-PLB (PLB-t, 1:3000, A010-13AP, Badrilla, United Kingdom), pSer^16^-PLB (PLB-s, 1:1000, GTX132818, Gene Tex, United States), sarcoplasmic reticulum (SR) Ca-ATPase (SERCA2a, 1:1000, MA3-919, Thermo Fisher Scientific), ryanodine receptor (RYR2, 1:2000, 19765-1-AP, BioTools, United States), and β-actin (1:20000, A5441, Sigma-Aldrich, United States) were used to detect the proteins of interest. Secondary antibodies, such as goat anti-mouse IgG-HRP (Leinco Technologies, United States), goat anti-rabbit IgG-HRP (Leinco Technologies), and donkey anti-goat IgG-HRP antibody (Santa Cruz Biotechnology, United States), were used in conjunction with primary antibodies. Signals were obtained through enhanced chemiluminescence (Pierce ECL Western Blotting Substrate, Thermo Fisher Scientific), and blots were quantified through scanning densitometry. The levels of protein expression were normalized to that of β-actin. One hundred points over the ventricular anterior wall were selected for APD analysis.

### Data Analysis

Action potential duration was measured at the level of 80% of repolarization. In each heart, the values of APD from the pixels of the mapped LV anterior wall were measured for calculating the mean APD. A computer algorithm automatically determined APD. We used global APD dispersion to estimate APD heterogeneity. We included all pixels in the mapped LV anterior epicardial surface in the calculation of the standard deviation of APD, which was used to represent the global APD heterogeneity index (Chang et al., 2018b). We used monoexponential fittings to compute the time constant tau (τ) value of the decay portion of the Ca_i_ transient between 70% of the transient peak and the diastolic baseline. The thresholds of spatially concordant alternans (SCA) of APD and Ca_i_ were defined as the longest PCL required to produce a 10-ms difference in APD and a 10% difference in Ca_i_ amplitude between consecutive beats, respectively. The phase was considered positive for a short–long APD and a small–large Ca_i_ amplitude sequence (color coded in red) and negative for a long–short APD and a large–small Ca_i_ amplitude sequence (color coded in green). SDA was evidenced by the presence of both red and green regions separated by a nodal line. The SDA threshold was defined as the longest PCL required to reach the alternans threshold on both sides of a nodal line. To estimate CV, we measured the distance and conduction time between the earliest activation point and two epicardial points in the non-IR (CV_non–IR_) and IR (CV_IR_) zones.

### Statistical Methods

Continuous variables are expressed as mean ± standard deviation, and categorical variables are represented by numbers and percentages. Repeated-measures ANOVA with *post hoc* LSD analysis was performed to analyze differences in ERP, CV, Ca_i_ decay, and the thresholds of SCA, SDA, and VF induction in the TH groups. Student’s *t*-test was performed to compare the above parameters in the non-TH group and CV, APD, and Ca_i_ decay between the non-IR and IR zones. Categorical variables were evaluated using Fisher’s exact test and *post hoc* Bonferroni analysis. Statistical analyses were performed using IBM SPSS V22.0 (Armonk, NY, United States). Differences were considered significant at *P* < 0.05.

## Results

After 4-week pacing, the LV ejection fraction was 38% ± 7% in the TH group and 37% ± 7% in the non-TH group (*P* = 0.74). No significant difference was observed in body weight (3.2 ± 0.3 kg vs. 3.3 ± 0.3 kg, *P* = 0.19) or dry heart weight (17.0 ± 1.8 g vs. 18.5 ± 1.9 g, *P* = 0.09) between the TH and non-TH groups. Spontaneous VF occurred in 4 of 14 rabbits in the TH group and 2 of 7 rabbits in the non-TH group during acute coronary artery ligation. These rabbits had a significantly lower LV ejection fraction than those with no spontaneous VF (33% ± 1% vs. 40% ± 7%, *P* = 0.049).

### Electrophysiological Effects of TH and Rotigaptide on Failing Hearts With Acute IR Injury

#### Effective Refractory Period and APD

**FIGURE 2:**
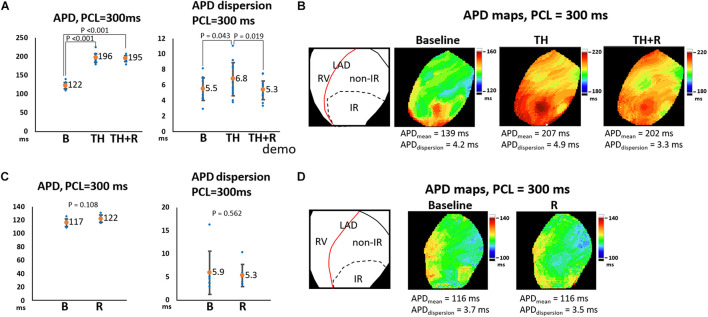
Effects of therapeutic hypothermia (TH) and rotigaptide (R) on action potential duration (APD) and APD dispersion (APD_dispersion_) at pacing cycle length (PCL) of 300 ms. **(A,C)** Summarized results of APD and APD_dispersion_ in the TH (*n* = 14, repeated-measures ANOVA with *post hoc* LSD analysis) and non-TH groups (*n* = 7, Student’s *t*-test), respectively. Numbers indicate the mean values of APD (APD_mean_). TH significantly prolonged APD and increased APD_dispersion_, which were attenuated by rotigaptide. **(B,D)** Representative APD_80_ and APD_dispersion_ maps in the TH and non-TH groups, respectively. IR, ischemia–reperfusion; LAD, left anterior descending artery; RV, right ventricle.

**FIGURE 3:**
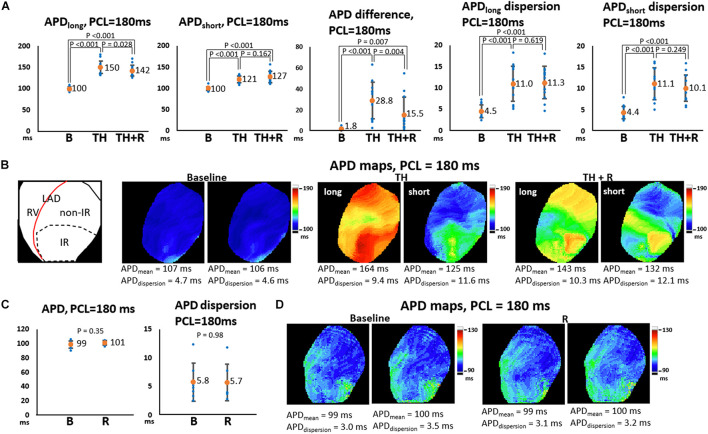
Effects of therapeutic hypothermia (TH) and rotigaptide (R) on action potential duration (APD) and APD dispersion (APD_dispersion_) at a pacing cycle length (PCL) of 180 ms. **(A,C)** Summarized results of APD, APD difference, and APD_dispersion_ in the TH (*n* = 14, repeated-measures ANOVA with *post hoc* LSD analysis) and non-TH groups (*n* = 7, Student’s *t*-test), respectively. Numbers indicate the mean values of APD (APD_mean_). Beat-to-beat APD variability was noted at shorter PCLs during TH. TH prolonged APD and increased APD difference and APD_dispersion_ significantly. Rotigaptide shortened the longer APD but did not change the shorter APD significantly, resulting in a decrease in APD difference between the consecutive beats. **(B,D)** Representative APD_80_ and APD_dispersion_ maps in the TH and non-TH groups, respectively. IR, ischemia–reperfusion; LAD, left anterior descending artery; RV, right ventricle.

In the TH group, instituting TH prolonged ERP (221 ± 16 ms vs. 139 ± 14 ms, *P* < 0.001) and APD (196 ± 12 ms vs. 122 ± 9 ms, *P* < 0.001) and increased APD dispersion significantly at PCL of 300 ms (6.8 ± 2.2 ms vs. 5.5 ± 1.5 ms, *P* = 0.043). Subsequent rotigaptide administration had no significant effects on ERP (222 ± 17 ms vs. 221 ± 16 ms, *P* = 0.43) or APD (195 ± 8 ms vs. 196 ± 12 ms, *P* = 0.83), but it decreased APD dispersion at PCL of 300 ms (5.3 ± 1.3 ms vs. 6.8 ± 2.2 ms, *P* = 0.019, Figure 2A) in the TH group. Figure 2B presents an example of APD maps at baseline (37°C), TH, and rotigaptide at PCL of 300 ms. TH prolonged APD (from 139 ms to 207 ms) and increased APD dispersion (from 4.2 ms to 4.9 ms). Rotigaptide did not significantly change APD (from 207 ms to 202 ms) but reduced APD dispersion (from 4.9 ms to 3.3 ms). In the non-TH group, rotigaptide had no significant effects on ERP (136 ± 14 ms vs. 134 ± 13 ms, *P* = 0.36), APD (122 ± 5 ms vs. 117 ± 6 ms, *P* = 0.11), or APD dispersion at PCL of 300 ms (5.3 ± 2.4 ms vs. 5.9 ± 4.6 ms, *P* = 0.56, Figure 2C). As presented in Figure 2D, rotigaptide made no discernible changes in APD (from 116 ms to 116 ms) or APD dispersion (from 3.7 ms to 3.5 ms) at PCL of 300 ms in this representative heart. At PCL of 180 ms, APD alternans was induced during TH. As presented in Figure 3A, TH prolonged APD [150 ± 4 ms vs. 100 ± 1 ms, P < 0.001 for the longer APD (APD_long_) and 121 ± 2 ms vs. 100 ± 1 ms, *P* < 0.001 for the shorter APD (APD _short_)] and increased beat-to-beat APD difference (28.8 ± 4.8 ms vs. 1.8 ± 0.6 ms, *P* < 0.001) compared to the baseline. Rotigaptide shortened APD_long_ (142 ± 4 ms vs. 151 ± 4 ms, *P* = 0.028) but did not significantly change APD_short_ (127 ± 4 ms vs. 121 ± 2 ms, *P* = 0.162), resulting in a decrease in beat-to-beat APD difference (15.5 ± 4.5 ms vs. 28.8 ± 4.8 ms, *P* = 0.004). Figure 3B presents an example of APD maps at baseline, TH, and rotigaptide at PCL of 180 ms.

Beat-to-beat variability of APD was noted during TH (164 ms vs. 125 ms, APD difference = 39 ms), which was reduced after rotigaptide administration (143 ms vs. 132 ms, APD difference = 11 ms). In the non-TH group, rotigaptide had no significant effects on APD (101 ± 3 ms vs. 99 ± 5 ms, *P* = 0.35), or APD dispersion at PCL of 180 ms (5.7 ± 3.2 ms vs. 5.8 ± 3.4 ms, *P* = 0.98, Figure 3C). As presented in Figure 3D, no significant beat-to-beat variability of APD was noted at baseline and with rotigaptide, and rotigaptide made no discernible changes in APD or APD dispersion at PCL of 180 ms in this representative heart.

#### Intracellular Ca^2+^ Decay

**FIGURE 4:**
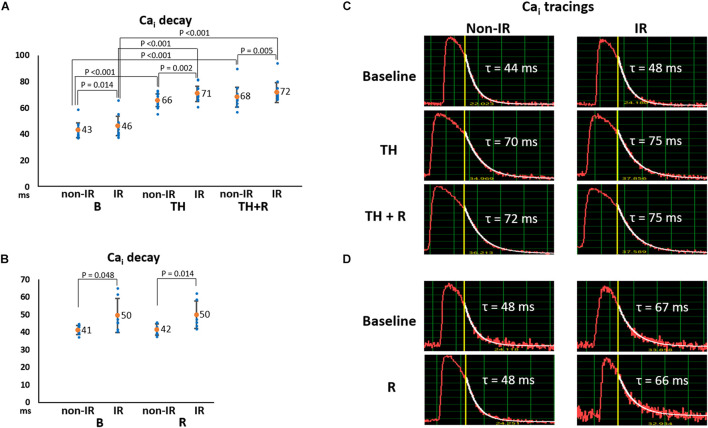
Effects of therapeutic hypothermia (TH) and rotigaptide (R) on intracellular Ca^2+^ (Ca_i_) decay. **(A,B)** Summarized results of Ca_i_ decay tau (τ) values between the ischemia–reperfusion (IR) and non-IR zones in the TH (*n* = 14, repeated-measures ANOVA with *post hoc* LSD analysis) and non-TH groups (*n* = 7, Student’s *t*-test), respectively. Numbers indicate the mean values. TH significantly increased the τ values in both non-IR and IR zones. Rotigaptide had no significant effects on Ca_i_ decay. **(C,D)** Representative examples of Ca_i_ decay in the non-IR and IR zones in the TH and non-TH groups, respectively.

Intracellular Ca^2+^ decay is an indicator of SR Ca^2+^ uptake. As presented in Figures 4A,B, Ca_i_ decay time (τ value) was significantly longer in the IR zone than in the non-IR zone at all stages. TH prolonged Ca_i_ decay time in the IR (71 ± 6 ms vs. 46 ± 7 ms, *P* < 0.001) and non-IR zones (66 ± 5 ms vs. 43 ± 6 ms, *P* < 0.001, Figure 4A) compared to the baseline. Rotigaptide had no significant effect on Ca_i_ decay. Figure 4C presents an example of Ca_i_ tracings in the TH group. The τ values were increased in the IR and non-IR zones during TH and were not changed significantly by rotigaptide. Similarly, rotigaptide did not significantly change the τ values in the non-TH group (Figure 4B), and a representative example is shown in Figure 4D.

#### Conduction Velocity

**FIGURE 5:**
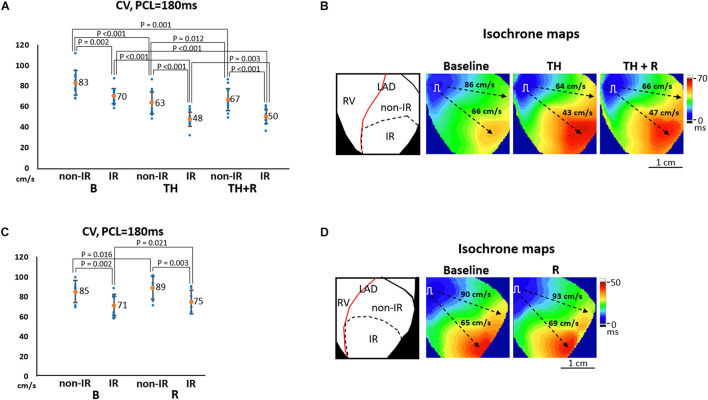
Effects of therapeutic hypothermia (TH) and rotigaptide (R) on conduction velocity (CV). **(A,C)** Summarized results of CV at pacing cycle length (PCL) of 180 ms in the TH (*n* = 14, repeated-measures ANOVA with *post hoc* LSD analysis) and non-TH groups (*n* = 7, Student’s *t*-test), respectively. TH slowed CV and rotigaptide ameliorated conduction slowing in both ischemia–reperfusion (IR) and non-IR zones. **(B,D)** Representative examples of isochronal maps in the TH and non-TH groups, respectively. Dashed black arrows indicate the directions of wavefront propagation.

Figure 5A summarizes the effects of TH and rotigaptide on CV at PCL = 180 ms in the TH group. CV_non–IR_ was faster than CV_IR_ at all stages. TH significantly slowed CV_non–IR_ (63 ± 10 ms vs. 83 ± 12 ms, *P* < 0.001) and CV_IR_ (48 ± 7 ms vs. 70 ± 8 ms, *P* < 0.001), which were ameliorated by rotigaptide (67 ± 11 ms vs. 63 ± 10 ms, *P* = 0.012 for CV_non–IR_; and 50 ± 7 ms vs. 48 ± 7 ms, *P* = 0.003 for CV_IR_). Note that the attenuation of TH-induced conduction slowing by rotigaptide was incomplete as there was still a significant difference compared to the baseline. In addition, three of 14 hearts exhibited no discernible change in CV after rotigaptide administration. Figure 5B illustrates a representative example. TH decreased CV_non–IR_ and CV_IR_ from 86 cm/s to 64 cm/s and from 66 cm/s to 43 cm/s, respectively, which were increased to 66 cm/s and 47 cm/s by rotigaptide, respectively. Similarly, rotigaptide administration increased CV_non–IR_ (89 ± 12 ms vs. 85 ± 11 ms, *P* = 0.016) and CV_IR_ (75 ± 12 ms vs. 71 ± 12 ms, *P* = 0.021) in the non-TH group (Figure 5C), and one of seven hearts showed no discernible change in CV after rotigaptide. A representative example is presented in Figure 5D.

#### Thresholds of SCA and SDA

**FIGURE 6:**
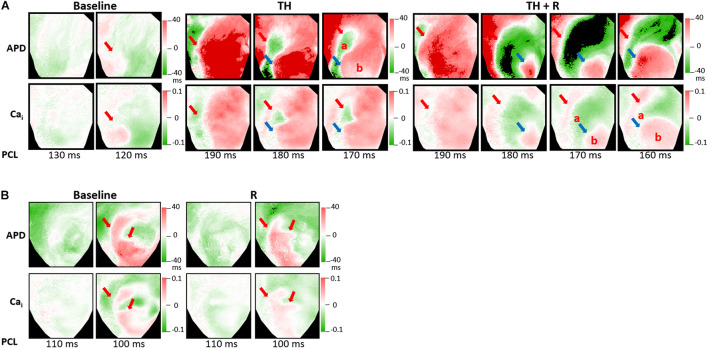
Effects of therapeutic hypothermia (TH) and rotigaptide (R) on spatially discordant alternans (SDA) induction by dynamic pacing. **(A)** Representative examples of action potential duration (APD) and intracellular Ca^2+^ (Ca_i_) alternans maps in the TH group. TH enhanced SDA. The second nodal line gradually moved to the border of the IR zone when the heart was paced at a shorter pacing cycle length (PCL). Red and blue arrows indicate the first and second nodal lines. **(B)** Representative examples of APD and intracellular Ca_i_ alternans maps in the non-TH group.

Alternans represents a phenomenon of electrophysiologic instability. SDA is the phenomenon of further heterogeneity of conduction and Vm/Ca_i_ homeostasis, leading to functional conduction block and arrhythmias. In the TH group, SCA could be provoked in all hearts. TH lowered SCA threshold (254 ± 34 ms vs. 139 ± 21 ms, *P* < 0.001), and rotigaptide elevated SCA threshold significantly (246 ± 36 ms vs. 254 ± 34 ms, *P* = 0.028). Moreover, SDA could be induced in all hearts. TH lowered the SDA threshold (195 ± 27 ms vs. 111 ± 12 ms, *P* < 0.001). Rotigaptide did not significantly elevate the SDA threshold (190 ± 21 ms vs. 195 ± 27 ms, *P* = 0.236), but deferred the movement of nodal lines toward the pacing site during dynamic pacing. Figure 6A presents a representative example of SDA induction in the TH group. At baseline, SDA was not induced until PCL decreased to 120 ms. During TH, SDA was provoked at PCL = 190 ms. Note that the second nodal line (blue arrow) was already located at the border of the IR zone at PCL = 180 ms. After rotigaptide administration, SDA could be provoked at PCL = 190 ms, but the second nodal line was still away from the border of the IR zone at PCL = 180 ms. When the heart was paced at shorter PCLs, the second nodal line gradually moved to the pacing site (the left upper corner of the map) and came to the border of the IR zone at PCL = 160 ms. This phenomenon was observed in eight of 14 hearts in which a shortening of PCL (by 13 ± 5 ms) was required to achieve the same pattern of TH-induced SDA. In the non-TH group, SCA and SDA were induced in seven and six hearts, respectively. Rotigaptide significantly decreased the SCA threshold (144 ± 24 ms to 136 ± 17 ms, *P* = 0.045) but neither the threshold (110 ± 10 ms vs. 108 ± 8 ms, *P* = 0.373) nor the pattern of SDA was significantly changed by rotigaptide. A representative example of SDA induction in the TH group is presented in Figure 6B.

#### Ventricular Fibrillation Inducibility

**FIGURE 7:**
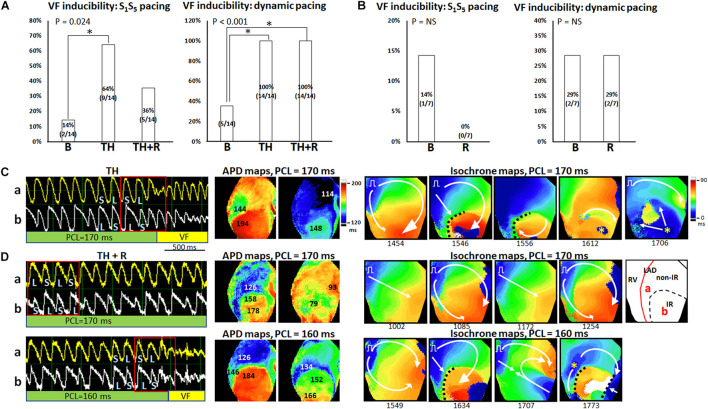
Effects of therapeutic hypothermia (TH) and rotigaptide (R) on ventricular fibrillation (VF) induction. **(A,B)** Summarized results of VF inducibility in the TH (*n* = 14, Fisher’s exact test and *post hoc* Bonferroni analysis) and non-TH groups (*n* = 7, Fisher’s exact test), respectively. A single asterisk indicates *P* < 0.05. **(C)** A representative example of VF induction during TH of the same heart in Figure 6A. Left: Vm tracing showing VF initiation at PCL = 170 ms in the TH group. S, short; L, long. Middle: APD maps showing large APD gradients at the sites of conduction block. Right: isochrone maps of VF initiation corresponding to Vm tracing labeled by a red square. **(D)** VF induction after rotigaptide administration. Upper: At PCL of 170 ms, Vm alternans was out-of-phase between site a and site b. APD maps showed smaller APD gradients at the sites of conduction block. Isochrone maps show the beat-to-beat alternation of conduction velocity. Right subpanel: schematic of the mapping field. Lower: VF induction at PCL of 160 ms. Large APD gradients were induced at the sites of conduction block. White arrows indicate the directions of wavefront propagation; dashed black lines indicate the functional conduction block; yellow asterisks, epicardial breakthroughs.

Figures 7A,B summarizes the VF inducibility by the extrastimulus pacing and dynamic pacing protocols in the TH and non-TH groups, respectively. In the TH group, through the extrastimulus pacing protocol, VF was induced in two, nine, and five hearts at baseline, TH, and rotigaptide, respectively (*P* = 0.024). The *post hoc* analyses revealed that VF inducibility was significantly higher in the TH group. Rotigaptide did not significantly reduce the TH-enhanced VF inducibility, but the difference in VF inducibility was also not significant between baseline and with rotigaptide. Through the dynamic pacing protocol, VF was induced in 5, 14, and 14 hearts (*P* < 0.001) at baseline, with TH, and with rotigaptide, respectively. The *post hoc* analyses revealed that TH significantly increased VF inducibility, which was not reduced significantly by rotigaptide. However, the VF threshold was significantly lowered by TH (166 ± 27 ms vs. 114 ± 13 ms, *P* = 0.008) and elevated by rotigaptide (154 ± 20 ms vs. 166 ± 27 ms, *P* = 0.026). That is, rotigaptide increased the pacing threshold of VF even if it did not significantly reduce VF inducibility by dynamic pacing. Figure 7C illustrates an example of VF induction during TH of the same heart in Figure 6A. The left subpanel shows the Vm tracings of VF induction by dynamic pacing at PCL = 170 ms. Note that SDA was provoked preceding VF induction. The development of SDA amplified repolarization gradients to produce functional conduction block. As presented in the APD maps (middle subpanels), a large APD gradient (50 ms) occurred at the sites of the conduction block. In the isochrone maps (right subpanels), consecutive short-coupling pacing beats caused functional conduction block (frame 1546), and a wavefront surrounded a nodal line to activate the IR zone (frame 1556), followed by breakthroughs (yellow asterisks, frame 1612) and multiple wavefronts (frame 1706) to initiate VF. Figure 7D presents an example of VF induction after rotigaptide administration. SDA was also provoked at PCL = 170 ms (left subpanel, upper), but VF was not induced until PCL was shortened to 160 ms (left subpanel, bottom). Consecutive short-coupling pacing at PCL = 170 ms induced CV alternans (fast CV in frames 1002 and 1172; slow CV in frames 1085 and 1254) but did not induce functional conduction block because the second nodal line (blue arrows, Figure 6A) was far away from the border of the IR zone. Also, note that the APD gradient (32 ms) was smaller with rotigaptide than that during TH at PCL of 170 ms (middle subpanels). When PCL was shortened to 160 ms, the APD gradient was increased to 58 ms at the sites of conduction block. The second nodal line moved to the border of the IR zone and functional conduction block was formed (frame 1634), followed by a wavefront from the right border of the mapping field (frame 1707) colliding with the pacing beat to generate multiple wavefronts initiating VF (frame 1773, right subpanels, bottom). In the non-TH group, VF was induced in one and zero heart by extrastimulus pacing (*P* = NS) and two and two hearts by dynamic pacing (*P* = NS) at baseline and with rotigaptide, respectively.

### Effects of TH on Protein Expression in IR and Non-IR Zones

**FIGURE 8:**
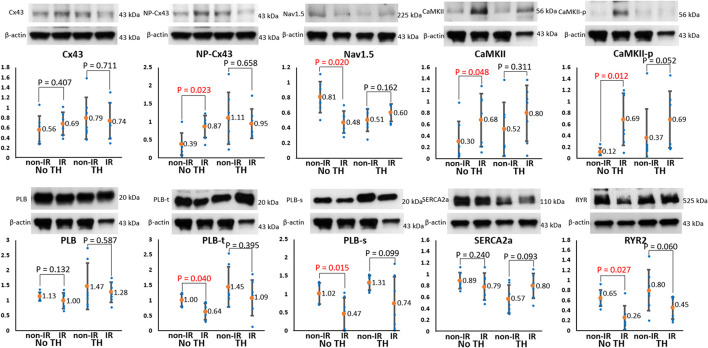
Results of protein analyses. Representative examples of channel proteins (upper subpanels) and summarized results of channel proteins (lower subpanels) (*n* = 6, Student’s *t*-test for all comparisons). Non-phosphorylated connexin43 (NP-Cx43), calmodulin-dependent protein kinase II (CaMKII), and pThr287-CaMKII (CaMKII-p) expression was significantly upregulated, and Nav1.5, pThr^17^-phospholamban (PLB-t), pSer^16^-phospholamban (PLB-s), and ryanodine receptor (RYR2) expression was significantly downregulated in the ischemia–reperfusion (IR) zone in the non-therapeutic hypothermia (TH) group but not in the TH group. Because of technical issues and various protein sizes, Western blots protein analyses were conducted at different gels. The illustrative examples of Cx43 and NP-Cx43 were acquired from no-TH heart 1 and TH heart 1; the examples of CaMKII, CaMKII-P, PLB, PLB-t, PLB-s were from no-TH heart 2 and TH heart 2; the examples of Nav1.5 were from no-TH heart 3 and TH heart 3; the examples of sarcoplasmic reticulum Ca-ATPase (SERCA2a) were from no-TH heart 4 and TH heart 4; the examples of ryanodine receptor (RYR2) were from no-TH heart 5 and TH heart 5.

To elucidate the roles of ion channels and Cx43 in arrhythmogenesis during TH, we measured and compared the levels of the associated proteins between the IR and non-IR zones. Figure 8 summarizes the results. In the non-TH group, we observed a significant upregulation of NP-Cx43 and a downregulation of Nav1.5 in the IR zone. These dysregulated alterations in protein expression were ameliorated in the TH group. In addition, CaMKII and CaMKII-p expression levels were higher and PLB-t, PLB-s, and RYR2 expression levels were significantly lower in the IR zone in the non-TH group, but not in the TH group. That is, TH attenuated the dysregulation of these proteins in the IR zone during acute IR injury. The expression levels of SERCA2a and total PLB did not differ significantly between the IR and non-IR zones in the two groups.

## Discussion

The main findings in this study are as follows: (1) In failing rabbit hearts with acute IR injury, TH increased APD dispersion and beat-to-beat variability, aggravated conduction slowing, and prolonged Ca_i_ decay to facilitate SCA, SDA, and VF induction; thus, TH is proarrhythmic; (2) however, TH attenuated IR-induced upregulation of NP-Cx43, CaMKII, and CaMKII-p and downregulation of Nav1.5, PLB-t, and PLB-s, which are supposed to be antiarrhythmic at the molecular level; (3) rotigaptide had no significant effects on APD, ERP, or Ca_i_ decay, but it reduced TH-induced APD dispersion and beat-to-beat variability, and ameliorated conduction slowing to elevate the SCA threshold, defer the onset of arrhythmogenic SDA, and elevate the pacing threshold of VF during TH. However, the effect of rotigaptide on TH-enhanced VF inducibility was statistically insignificant. In non-TH hearts, rotigaptide improved CV and raised SCA threshold but had no significant effects on APD dispersion, SDA threshold, or VF inducibility. The more obvious antiarrhythmic effect of rotigaptide on TH-treated hearts suggests a poorer gap junctional coupling during TH.

### Controversy of Cx43 Protein Remodeling in Conduction Slowing During TH in Failing Hearts With Acute IR Injury

The functional role of gap junction remodeling during TH is ambiguous. In a canine LV preparation model, Nassal et al. (2017) demonstrated that the hypothermic modulation of Cx43 maintained myocardial conduction and reduced susceptibility to conduction block during ischemia. However, Hsieh et al. (2011) reported that TH decreases NP-Cx43, which coincided with conduction disturbance in normal rabbit hearts. Cx43 dephosphorylation has been shown to promote conduction abnormalities and facilitate arrhythmias (Severs et al., 2004). Why a decrease in NP-Cx43 results in conduction slowing during TH remains unclear. Our study also showed similar results that TH attenuated NP-Cx43 upregulation in the IR zone, which coincided with aggravation rather than improvement of conduction slowing. Because the conductance of gap junctional membranes is temperature dependent (Bukauskas and Weingart, 1993), functional rather than structural remodeling of Cx43 may have accounted for the TH-induced conduction slowing. NP-Cx43 downregulation may be a compensatory response to maintain a sufficient gap junction coupling when TH aggravates gap junction uncoupling in the IR zone (Hsieh et al., 2011).

Beardslee et al. (2000) reported that Cx43 dephosphorylation and NP-Cx43 accumulation within gap junctions are measurable within the first hour of ischemia, but these responses are variable during reperfusion. Myocardial reperfusion aggravates Ca_i_ overload *via* reverse-mode Na^+^/Ca^2+^ exchanger to hinder the reaccumulation of phosphorylated Cx43 and gap junction coupling. However, partial restoration of the control level of phosphorylated Cx43 may occur in hearts with a recovery of contractile function during reperfusion (Beardslee et al., 2000). Our data showed that rotigaptide improved conduction slowing after IR injury, but three of 14 hearts in the TH group and one of seven hearts in the non-TH group exhibited no discernible changes in CV after rotigaptide treatment. A possible explanation is that the partial restoration of phosphorylated Cx43 during reperfusion blunted the action of rotigaptide and other mechanisms instead of Cx43 dysfunction accounted for conduction slowing in rotigaptide-unresponsive hearts.

Our results also reveal that rotigaptide improved CV_IR_ and CV_non–IR_ in both TH and non-TH hearts. Heart failure is associated with a heterogeneous reduction in Cx43 expression, potentially resulting in slowed conduction (Dupont et al., 2001). Liu et al. (2014) reported that rotigaptide reduces vulnerability to ventricular arrhythmias in failing rabbit hearts with downregulation of Cx43 mRNA. In addition to TH and IR injury, preexisting heart failure may account partly for the general improvement of CV by rotigaptide in this model.

### Role of *I*_Na_ in Conduction Slowing in Failing Hearts With Acute IR Injury Undergoing TH

Our results reveal that IR induced downregulation of Nav1.5 and upregulation of CaMKII and CaMKII-p protein expression in the non-TH group. CaMKII is highly sensitive to cytosolic Ca^2+^ levels and susceptible to oxidation. Therefore, IR injury provides an optimal setting for CaMKII activation (Bell et al., 2014). Acute IR injury activates CaMKII to phosphorylate Nav1.5, leading to the loss-of-function changes in *I*_Na_ gating (Ashpole et al., 2012). Moreover, heart failure is associated with decreased *I*_Na_ and upregulated CaMKII (Ufret-Vincenty et al., 2001; Ai et al., 2005). In addition to gap junction uncoupling, *I*_Na_ dysfunction seems to play an important role in conduction disturbance in this model.

Similar to Cx43, our data show that TH attenuated IR-induced downregulation of Nav1.5 and upregulation of CaMKII and CaMKII-p protein expression, which coincided with aggravation rather than an improvement of conduction disturbance. Because TH may decrease *I*_Na_ availability through its temperature-dependent slowing of activation/inactivation kinetics (Kirsch and Sykes, 1987), functional rather than structural changes in the Nav1.5 channel likely account for TH-induced conduction slowing (Hsieh et al., 2011).

### Effects of Rotigaptide on APD Dispersion, Beat-to-Beat Repolarization Variability, and SDA in Failing Hearts With Acute IR Injury Undergoing TH

Hypothermia-induced cell-to-cell uncoupling enhances spatial APD dispersion and beat-to-beat variability, contributing to conduction slowing, and promoting the onset of SDA (Fedorov et al., 2008; Egorov et al., 2012). Increased dispersion of APD causes conduction failure locally and has long been known to facilitate VF (Kuo et al., 1983; Harada et al., 2011). Slow conduction is a requirement for the perpetuation of reentry, but unidirectional block and recovery of excitability distal to the site of block must occur for the initiation of reentry (Lesh et al., 1989). In a computer modeling study, Lesh et al. showed that the higher the coupling resistance between cells, the less the electrotonic interaction and the greater the unmasking of local differences in intrinsic APD. That is, the cellular coupling can electronically modulate APD and thus dynamically affect the local dispersion of refractoriness needed for conduction block (Lesh et al., 1989). Consistently, our data show large APD gradients occurred at the sites of conduction block at PCL of 170 ms during TH; rotigaptide infusion reduced APD gradients at the same PCL (Figure 7). TH significantly increased the global APD heterogeneity index from a mean of 5.5 ms to 6.8 ms, and rotigaptide reduced it to 5.3 ms at PCL of 300 ms. However, the differences were small, possibly because the global APD heterogeneity index was determined by the standard deviation of thousands of pixels over a large area. Furthermore, the increased dispersion of APD over a large area may be less likely to be arrhythmogenic. On the other hand, beat-to-beat variability of APD, which depends on stochastic ion channel gating, Ca_i_ handing, and intercellular coupling (Magyar et al., 2015), has been suggested to predict the development of lethal arrhythmias in patients with non-ischemic heart failure (Hinterseer et al., 2010). Bjørnstad et al. (1993) reported that hypothermia not only significantly lengthens APD but also magnifies the PCL-dependent shortening of APD to increase the beat-to-beat differences of APD at shorter PCLs. Zaniboni et al. (2000) demonstrated that the beat-to-beat repolarization variability could be suppressed by enhancing electrical coupling in isolated guinea pig ventricular myocytes. Consistently, our data show that rotigaptide significantly decreased the beat-to-beat differences in APD at PCL of 180 ms, which could account for, at least partly, the antiarrhythmic actions of rotigaptide in this model.

Ischemia–reperfusion, heart failure, and hypothermia induce gap junction uncoupling and are associated with a decreased upstroke velocity of the action potential, leading to slowed conduction and dispersion of repolarization (Bjørnstad et al., 1993; Kjølbye et al., 2002; Poelzing and Rosenbaum, 2004). Under these circumstances, cardiomyocytes distant from the pacing site experience a longer diastolic interval than cardiomyocytes close to the pacing sites. Therefore, in spite of short coupling intervals, cells distant from the pacing site may develop paradoxically longer APD, giving rise to SDA (Qu et al., 2000; Kjølbye et al., 2008). SDA can steeply increase the spatial APD gradient enough to cause functional conduction block and reentry arrhythmia (Pastore and Rosenbaum, 2000). Hsieh et al. reported that in normal rabbit hearts, rotigaptide decreased APD dispersion but did not change SDA threshold during TH. However, the susceptibility to pacing-induced VF was significantly decreased by rotigaptide (Hsieh et al., 2016). Why rotigaptide suppressed VF inducibility without affecting SDA threshold during TH was unclear in that study. We observed similar results in our study; rotigaptide reduced TH-induced APD dispersion and beat-to-beat variability and conduction slowing to elevate the pacing threshold of VF. Rotigaptide administration did not significantly affect the SDA threshold but deferred the movement of nodal lines toward the pacing site during dynamic pacing. Hayashi et al. reported that SDA can be formed *via* a dynamic pattern formation process; that is, nodal lines evolve toward a steady-state pattern that depends on pacing rate. These patterns originate far from the pacing site and move closer as the pacing rate increases (Hayashi et al., 2007). As presented in Figure 7, the second nodal line approaching the border of the IR zone played a pivotal role in inducing functional conduction block for VF initiation during TH, and a PCL shortened by 20 ms was needed for this movement after rotigaptide administration in this heart. SDA is a dynamic entity that depends on the CV and APD restitution, Ca_i_ dynamics, and underlying tissue heterogeneity (Chou et al., 2007; Hayashi et al., 2007). Restoration of electrical coupling increases CV and shortens the time differences in depolarization between cells, thereby diminishing the spatial gradients of activation and deferring the onset of arrhythmogenic SDA.

### Modulation of Ca_i_ During TH in Failing Hearts With Acute IR Injury

In addition to CaMKII upregulation, our data show that cardiac IR injury induced downregulation of PLB-t, PLB-s, and RYR2 protein expression. Downregulation of phosphorylated forms of PLB decreases SR Ca^2+^ uptake, which may explain slower Ca_i_ decay in the IR zone (Figure 4). Similar to Cx43 and *I*_Na_, TH attenuated the IR-induced downregulation of phosphorylated PLB but did not improve the slowing of Ca_i_ decay in the IR zone. Hypothermia retards the activation and/or inactivation process, leading to ion channel dysfunction. For example, Fukaya et al. showed that hypothermia caused Ca_i_ overload and slowing of SR Ca^2+^ release kinetics through RyR2 dysfunction in a temperature-dependent manner (Fukaya et al., 2017). Millet et al. (2021) reported that hypothermia retarded SERCA2a-mediated Ca^2+^ uptake into the SR to facilitate Ca_i_ alternans during tachycardia. Therefore, unsurprisingly, attenuation of IR-induced alterations in calcium-handling protein expression could not reflect its functional roles during TH in this study. Furthermore, both hypothermia-induced RYR2 and SERCA2a dysfunction promote the emergence of SDA (Wang et al., 2021). Because rotigaptide has no significant effects on Ca_i_ homeostasis, the modulation of Ca_i_ dynamics, for example, direct or indirect CaMKII inhibition (Mitsuyama et al., 2014; Chou et al., 2020a), may work synergistically with rotigaptide to suppress arrhythmogenic alternans in our model. Further investigation is required to verify this hypothesis.

### Study Limitations

A limitation of the study is that there was no non-heart failure group. Therefore, the overall impact of rotigaptide use on the non-heart failure population can’t be assessed. Given that the cardiac IR model was created *in vivo* but optical mapping studies were performed in isolated Langendorff-perfused hearts, TH had to be instituted after reperfusion. Even though a previous study showed that delayed treatment with hypothermia protects against no-reflow phenomenon, thereby decreasing infarct expansion and reperfusion injury (Hale et al., 2013), most experimental studies have suggested that earlier-onset TH results in greater protection. Therefore, the effects of TH and rotigaptide may be different when TH is instituted before reperfusion and when the hearts are *in vivo*. Pretreatment of rotigaptide may prevent dephosphorylation of Cx43 during ischemia (Kjølbye et al., 2008). Because the protein expression data were all performed after rotigaptide administration, it is possible that rotigaptide played a role in attenuating IR-induced upregulation of NP-Cx43, CaMKII, and CaMKII-p and downregulation of Nav1.5, PLB-t, and PLB-s in the TH group. However, we did not observe that rotigaptide exerted similar effects on protein expression in the non-TH group. Possibly the duration of rotigaptide perfusion was too short (around 28 min) to modulate protein expression significantly in this study. The protocol for the TH group was longer than for the non-TH group by 28 min. We do not know whether the extra 28 min of hypothermia was a significant confounding factor that interfered with the interpretation of protein expression. We calculated the standard deviation of thousands of pixels in the mapped LV anterior epicardial surface to represent APD heterogeneity. However, there is not a definite “abnormal” APD heterogeneity index level regarding arrhythmia risk for now. Furthermore, the approach differences in mapped area between hearts are not accounted for.

## Data Availability Statement

The raw data supporting the conclusions of this article will be made available by the authors, without undue reservation.

## Ethics Statement

The animal study was reviewed and approved by Institutional Animal Care and Use Committee of Chang Gung Memorial Hospital, Taiwan (approval no. 2018090301).

## Author Contributions

H-LL performed optical mapping experiments, analyzed data, and wrote manuscript. P-CC performed pacemaker implantation and optical mapping experiments. H-TW performed immunoblotting and data analyses. H-TL performed pacemaker implantation and immunoblotting. M-SW contributed to study design and manuscript revision. C-CC contributed to study design, optical mapping experiments, data analyses, and manuscript writing and revision. All authors contributed to the article and approved the submitted version.

## Conflict of Interest

The authors declare that the research was conducted in the absence of any commercial or financial relationships that could be construed as a potential conflict of interest.

## Publisher’s Note

All claims expressed in this article are solely those of the authors and do not necessarily represent those of their affiliated organizations, or those of the publisher, the editors and the reviewers. Any product that may be evaluated in this article, or claim that may be made by its manufacturer, is not guaranteed or endorsed by the publisher.
